# Acceptability of Online Yoga Among Individuals With Chronic Conditions and Their Caregivers: Qualitative Study

**DOI:** 10.2196/39158

**Published:** 2023-05-24

**Authors:** Jennifer Dickman Portz, Arlene Schmid, Christine Fruhauf, Aimee Fox, Marieke Van Puymbroeck, Julia Sharp, Heather Leach

**Affiliations:** 1 University of Colorado Aurora, CO United States; 2 Colorado State University Fort Collins, CO United States; 3 Kansas State University Manhattan, KS United States; 4 Clemson University Clemson, SC United States

**Keywords:** yoga, meditative movement, online, chronic conditions, self-management, caregiver, dyads, meditation, participation, perspective, intervention

## Abstract

**Background:**

The online delivery of yoga interventions rapidly expanded during the COVID-19 pandemic, and preliminary studies indicate that online yoga is feasible across multiple chronic conditions. However, few yoga studies provide synchronous online yoga sessions and rarely target the caregiving dyad. Online chronic disease management interventions have been evaluated across conditions, life spans, and diverse samples. However, the perceived acceptability of online yoga, including self-reported satisfaction and online delivery preferences, is underexplored among individuals with chronic conditions and their caregivers. Understanding user preferences is essential for successful and safe online yoga implementation.

**Objective:**

We aimed to qualitatively examine the perceived acceptability of online yoga among individuals with chronic conditions and their caregivers who participated in an online dyadic intervention that merged yoga and self-management education to develop skills (MY-Skills) to manage persistent pain.

**Methods:**

We conducted a qualitative study among 9 dyads (>18 years of age; individuals experiencing persistent moderate pain) who participated in MY-Skills online during the COVID-19 pandemic. The intervention consisted of 16 online, synchronous yoga sessions over 8 weeks for both dyad members. After the completion of the intervention, participants (N=18) participated in semistructured telephone interviews for around 20 minutes, discussing their preferences, challenges, and recommendations for improved online delivery. Interviews were analyzed by using a rapid analytic approach.

**Results:**

MY-Skills participants were, on average, aged 62.7 (SD 19) years; were primarily women; were primarily White; and had a mean of 5.5 (SD 3) chronic conditions. Both participants and caregivers reported moderate pain severity scores (mean 6.02, SD 1.3) on the Brief Pain Inventory. The following three themes were identified related to online delivery: (1) participants indicated a preference for the intervention to be in person rather than online because they were distracted in the home setting, because they felt that in-person yoga would be more engaging, because the yoga therapist could physically correct positions, and because of safety concerns (eg, fear of falling); (2) participants indicated good acceptability of online MY-Skills delivery due to convenience, access, and comfort with being in their home; and (3) recommendations for improving online delivery highlighted a need for additional and accessible technical support.

**Conclusions:**

Both individuals with chronic conditions and their caregivers find online yoga to be an acceptable intervention. Participants who preferred in-person yoga did so due to distractions in the home and group dynamics. Some participants preferred in-person corrections to ensure correct positioning, while others felt safe with verbal modifications in their homes. Convenience and access were the primary reasons for preferring online delivery. To improve online delivery, future yoga studies should include specific activities for fostering group engagement, enhancing safety protocols, and increasing technical support.

**Trial Registration:**

ClinicalTrials.gov NCT03440320; https://clinicaltrials.gov/ct2/show/NCT03440320

## Introduction

Digital health technology has the potential to increase access to complementary and integrated health interventions for individuals with chronic conditions and their caregivers, including meditative movement approaches such as yoga [1,2]. The COVID-19 pandemic resulted in the increased awareness and uptake of digital health, including telehealth, mobile apps, and patient portals [3]. During this time, many yoga programs and research studies moved from in-person formats to online formats. Several online yoga studies that launched during the early pandemic targeted youth and specific medical illnesses, including cancer and posttraumatic stress [4-6]. Participants across studies report health and social benefits to participation in online yoga. However, understanding facilitators and barriers to online yoga delivery among individuals with chronic conditions is needed to sustain the current growth in digital health uptake.

Preliminary studies indicate that online yoga is feasible across multiple chronic conditions [7] and may benefit the 51.8% of Americans managing 1 chronic condition [8]. In addition, an estimated 65.7 million caregivers provide care to family members or friends and are at increased risk of chronic disease [9], and 41% of caregivers report having at least 2 chronic conditions [10]. However, few yoga studies provide synchronous online yoga sessions and rarely target the caregiving dyad. A recent review of remote yoga calls for additional attention toward online delivery methods during intervention design [11]. Examining the perceptions on online yoga among individuals with chronic conditions and their caregivers can inform improved remote delivery.

Although online chronic disease management interventions have been evaluated across conditions, life spans, and diverse samples [12], the perceived acceptability of online yoga, including self-reported satisfaction and online preferences, is underexplored among individuals with chronic conditions and their caregivers. Understanding user preferences is essential for successful and safe online yoga implementation [13]. Therefore, this paper aims to describe participant and caregiver perspectives regarding the acceptability of delivering a dyadic, online intervention that merges yoga and self-management education to develop skills (MY-Skills) to manage symptoms of chronic (termed herein as *persistent*) pain. Our objective for this paper is to qualitatively examine the acceptability of the online yoga sessions offered during MY-Skills and preferences for online yoga delivery.

## Methods

### Overview

We used a descriptive qualitative approach to analyze participants’ feedback, focusing on elements of online participation in yoga sessions [14-16]. After participation in the online MY-Skills intervention feasibility randomized controlled pilot, participants shared their overall experiences with the intervention in one-on-one interviews (Schmid et al, unpublished data, 2023). The primary qualitative analysis for the pilot focused on the perceived benefits of dyadic treatment for persistent pain [17]. This analysis targeted feedback solely related to online delivery.

### MY-Skills Feasibility Pilot

The primary goal of the pilot was to test the feasibility of MY-Skills among dyads experiencing persistent pain. MY-Skills is a standardized and progressive intervention that offered yoga and health education in 120-minute sessions (2 times per week for 8 weeks) via Zoom (Zoom Video Communications Inc) videoconferencing from October 2020 to June 2021. The yoga component was based on our prior studies [18] and was further developed specifically for dyads with persistent pain (Multimedia Appendix 1). The education component was based on literature regarding the self-management of persistent pain [19] and on stakeholder engagement [20]. Sessions included guided discussions, reflective activities, and brainstorming to address the common areas that are necessary in self-management education—problem-solving, action plans, the development of coping skills, and effective communication [21,22]. We included information about yoga for pain and a weekly mantra that was related to the education topic to merge the yoga and education sessions seamlessly. Yoga was delivered by a yoga therapist, and group education was provided by trained research assistants who identified as therapists (ie, occupational therapist or marriage and family therapist). Participants randomized to the control group participated in online, low-intensity exercise and health education that excluded yoga practice (ie, breath work and meditation) and self-management strategies (eg, goal setting, problem-solving, and health tracking).

### Ethics Approval

The Colorado State University Institutional Review Board approved all procedures (protocol number: 19-9095H). All participants were consented to participate via Zoom videoconference. Potential participants were provided with an e-consent form via REDCap (Research Electronic Data Capture; Vanderbilt University). The research assistant reviewed the consent form, answered any questions, and assessed participants’ understanding of this study. The participants and research assistant e-signed the consent form. All participants consented to this study prior to assessment and intervention. Participants received a US $50 Amazon e-card before and after intervention assessments and were provided with a yoga mat, blocks, and other props as needed. Data were captured via REDCap, Zoom, and phone; secured by using study IDs; and stored on servers behind university firewalls. All study personnel were trained in the conduct of human subject research.

### Recruitment

We recruited dyads (ie, caregivers and care receivers) with persistent pain from a local pain management clinic and through advertisements in the local newspaper, flyers, and community partners' newsletters and websites. To participate, both members of the dyad were required to be older than 18 years, report chronic musculoskeletal pain, and experience moderate or worse pain severity and pain interference (based on a score of >5 on the Brief Pain Inventory [23]). Additional inclusion criteria were reported elsewhere [24]. After the completion of the pilot, all participants (N=18) were invited to share their experiences in an interview. All participants agreed, and all completed the interview.

### Data Collection

Within 2 weeks after completing the MY-Skills intervention, we conducted one-on-one telephone interviews to acquire feedback from participants regarding dyadic experiences, perceived benefits of the intervention, and online delivery. Master- and doctoral-level research assistants with previous qualitative research experience used a semistructured interview guide that included questions about online intervention acceptability (Textbox 1). Before interviews, study investigators (CF and JP) conducted mock interviews with the team to prepare for data collection. Interviews were conducted with individuals separately or as a dyad, depending on participants’ preferences, and took around 20 minutes to complete.

**Interview guide example questions targeting online delivery.**

**Questions**

- “What was it like for you to complete the intervention online?”
- “Do you have a preference for online or face-to-face intervention?”
- “What challenges did you have completing the yoga/exercise in an online format?”
- “Did you feel safe completing the yoga/exercise on your own with only the online guidance of the facilitators?”
- “What could we have done to improve the online yoga/exercise?”
- “Do you have any additional comments, concerns, questions (including those about the online format and delivery)?”

### Analysis

We used rapid, qualitative, analytic methods to identify themes related to the acceptability of online delivery [25]. Interviews were audio recorded, interview summary notes were used for coding, and we revisited audio recordings to transcribe exemplar quotes for each code. Field notes from the time of the interviews were first entered into REDCap and exported to Excel (Microsoft Corporation) for coding. Field notes were coded (AF) and reviewed by the investigator team (JP, CF, and MVP). After codes were agreed upon by the team, exemplar quotes from the recordings were added and organized in Excel. Codes focused on the impact of MY-Skills on the dyad relationship, benefits experienced by participants, and feasibility feedback. For this paper, we focused solely on codes pertaining to the acceptability of online versus in-person delivery. First, JP reviewed online acceptability codes and collapsed codes into broad categories, which were then discussed with secondary coders (AF and MVP). To form overall findings, we conducted a heading and subheading thematic analysis, focusing on delivery preferences and recommendations for improved online delivery [26].

## Results

### Participants

A total of 16 dyads (N=32) enrolled in the online trial, and 9 dyads (n=18) were randomized to the MY-Skills group. MY-Skills participants were, on average, aged 62.7 (SD 19) years; were primarily women; were primarily White; and had a mean of 5.5 (SD 3) chronic conditions (Table 1). Both participants and caregivers reported moderate pain severity scores (mean 6.02, SD 1.3) on the Brief Pain Inventory.

**Table 1:** Caregiver and care receiver characteristics (N=18).^a^

		Caregivers (n=9)	Care receivers (n=9)
Age (years), mean (SD; range)		60.33 (20.72; 31-90)	65.00 (18.97; 27-86)
**Sex, n (%)**			
	Male	3 (33)	1 (11)
	Female	6 (67)	8 (89)
**Relationship status, n (%)**			
	Partnered	4 (44)	5 (56)
	Not partnered	5 (56)	4 (44)
**Race, n (%)**			
	Black	1 (11)	1 (11)
	White	7 (78)	8 (89)
	Other	1 (11)	0 (0)
**Ethnicity, n (%)**			
	Hispanic or Latinx	1 (11)	1 (11)
	Not Hispanic or Latinx	7 (78)	7 (78)
	Other or no answer	1 (11)	1 (11)
**Education, n (%)**			
	Some high school	0 (0)	0 (0)
	High school graduate	0 (0)	1 (11)
	Some college	3 (33)	4 (44)
	College graduate	6 (67)	4 (44)
**Baseline Brief Pain Inventory score, mean (SD)**			
	Pain severity	6.00 (1.48)	6.03 (1.11)
	Pain interference	5.65 (2.49)	6.08 (2.03)

^a^Frequencies may not sum to 100% due to unreported or missing data.

### Participant Perspectives of Online Yoga Delivery

Overall, participants’ responses indicated good acceptability of online MY-Skills delivery. Participants specified benefits for both the online format and the in-person format and provided several recommendations for improving online delivery (Textbox 2).

Participants enjoyed the online delivery of MY-Skills due to easy access and convenience. Many participants experienced transportation concerns related to their chronic conditions or distance to health care. The online option allowed individuals to participate regularly. Participants also appreciated online delivery because they felt safe and comfortable in their home environment. Participants indicated that instructors were able to modify and adjust individuals verbally as needed.

Some participants explained that in-person yoga would also have potential benefits. For example, practicing yoga in their home was occasionally distracting. Therefore, participating in person would encourage them to leave their home, allowing them to better focus on yoga practice. Other participants suggested that pose corrections would likely be easier in person and would make them feel safer when trying new poses. Several participants also suggested that in-person options would be more engaging, allowing for improved group interactions and dynamics.

To improve online delivery, participants recommended providing additional technical support and training. Several participants experienced issues with Zoom and internet connectivity; therefore, participants wanted more training to properly prepare for online sessions. Additional aids, such as manuals and videos with step-by-step instructions for connecting, muting, and using the chat function, among others, were also suggested for troubleshooting. In addition to technical support, participants recommended that more time be spent setting up their home environment for yoga practice.

**Summary of participant feedback and salient quotes.**

**In-person yoga preference**

- Home distractions
  “Would prefer face-to-face because of the distraction of watching the children during sessions and not being able to get the full benefit.” (Caregiver 4_07)
- Movement corrections
  “I would like to do it face-to-face to get the complete moves and make sure I am doing it 100% right.” (Care Receiver 2_04)
- Engagement
  “My preference would be face-to-face but that's not a possibility so I enjoyed what I got. I thought it was easy but I like the personal aspect of in person.” (Care Receiver 6_12)
- Safety concerns
  “We were a little concerned with more complicated exercises because we want the instructor to be able to make sure I was doing the exercises right to make sure I wouldn't get hurt.” (Care Receiver 7_14)

**Online yoga preference**

- Transportation or driving
  “It worked better for me because of my inability to travel.” (Care Receiver 1_02)
- Convenient
  “I really liked the online format. It was fun and engaging and also much more convenient than a face to face thing would have been.” (Caregiver 12_2)
  “Thought the online was very good. For me to be face to face with this group of people, it would never happen, but doing it online was quite interesting and enjoyable. Parking was a breeze!” (Caregiver 14_2)
- Comfort of home environment
  “I really liked it online, I think I would prefer it online-way more accessible and when you can take a break, it's really a break (because you're at home in a calm environment).” (Care Receiver 13_2)
- Felt safe
  “The instructor was amazing at noticing if anything was going wrong and stepping in. I felt very safe with it.” (Care Receiver 8_16)

**Recommendations for improving yoga for MY-Skills online**

- Technology access and support
  “Verify participants' internet access/compatibility with Zoom prior to starting intervention. A little bit of Zoom training prior to, to practice and become familiar with Zoom before starting.” (Caregiver 1_1)
- Improve internet connection
  “I think the main challenge was connection issues sometimes.” (Caregiver 13_2)
- Additional visual aids
  “Visual aids for the exercises itself. If there was an illustration of what muscle groups I was meant to tackle, that would've been helpful.” (Caregiver 11_2)
- Home space and setup
  “The first day that we did it, we didn't really set up a stable platform in our living room so it was a little shaky but we corrected it by putting some particle board down-stabilized the floor so we could exercise which turned out today.” (Care Receiver 11_2)

## Discussion

Our findings indicate that both individuals with chronic conditions and their caregivers found MY-Skills online yoga acceptable. Perceived health benefits and additional feasibility analyses of the pilot are currently under review elsewhere. Participants expressed preferences for either online yoga or in-person yoga and provided recommendations for improving online delivery. Leveraging these lessons learned may improve future MY-Skills and yoga studies.

Participants who preferred in-person yoga did so due to distractions in the home and group dynamics. Similarly, in a study (N=152) that compared in-person yoga conducted before the COVID-19 pandemic to online yoga conducted during the pandemic, in-person yoga participants provided higher ratings for feeling connected to other people and socializing before and after class [27]. Increasing the number of specific activities that foster group engagement and interaction may improve online acceptability [28]. These activities may include additional time or platforms for group icebreakers, dyadic team building, and opportunities for discussion or networking.

Participants preferred both online yoga and in-person yoga for different safety reasons. Some participants chose in-person corrections to ensure correct positioning, while others felt safe with verbal modifications in their homes. Although adverse events are rare in yoga research [29], online delivery poses additional challenges if a participant falls or injures themself. For MY-Skills, a caregiver and care receiver participated together as a dyad and could act as each other’s “safety net” if an issue arose. Future research is needed to standardize safety protocols for online yoga, particularly among individuals with chronic conditions. Creative solutions combining in-person and remote options, such as an initial in-person yoga therapy session followed by online group formats, may also maximize safety and intervention engagement and warrant further investigation [30,31].

Convenience and access were the primary reasons for preferring online delivery. This is similar to previous telehealth research [32,33]. However, barriers such as technical difficulties, device malfunctions, and connectivity are also well documented [34]. Recommendations for improving videoconference interventions include providing individual technical coaching, accessing stable internet and backups, and adapting technical support based on the needs of the participants [35].

Although this is one of the few studies that describe participants' reported acceptability of and preferences for online yoga, several limitations exist. Our sample is homogenous, indicating that these findings may not represent the perspectives of care receivers and caregivers from diverse backgrounds. Further, the lack of transcription may have resulted in additional bias in coding participant feedback. However, we followed standard, rapid, analytic techniques, such as group coding and discussion, to mitigate this concern. In addition, we did not collect validated measures of intervention usability or acceptability that could have contributed to a greater understanding of online acceptability.

In conclusion, participants find MY-Skills online yoga to be an acceptable intervention. To improve online delivery, future yoga studies should include specific activities for fostering group engagement, enhancing safety protocols, and increasing technical support.

## Appendix

Multimedia Appendix 1 Summary of online yoga sessions.

## Abbreviations

REDCap: Research Electronic Data Capture

## Data Availability

The data sets generated during and analyzed during this study are available from the corresponding author upon reasonable request.
