# Supplementary material for: Acceptability of Online Yoga Among Individuals With Chronic Conditions and Their Caregivers: Qualitative Study
Source: JMIR Form Res. 2023 May 24;7:e39158. doi: 10.2196/39158 (PMC10248766; doi:10.2196/39158)
Supplement: Multimedia Appendix 1 [file formative_v7i1e39158_app1.docx]

**Multimedia Appendix 1**

**Summary of Yoga Sessions**

| **Week** | **Yoga Poses and Breathwork** |
| --- | --- |
| 1 | **All seated**   - Grounding (Centering) – connect to mantra - Intro to pranayama (breathwork) - Spinal twist and axial extension (spinal movement) - Head and neck and eye movements with prolonged holds - Receptive gesture, cactus arms (shoulder flexion, shoulder extension, shoulder rolls, and arm movements) - Mudras (finger movements) - Forward fold (forward flexion) - Half moon (lateral flexion) - Seated savasana (body scan with progressive relaxation) |
|  |  |
| 2 | Last session plus:   - Grounding (Centering) – connect to mantra and body scan - Pranayama (breathwork) - Ankle rotation - Move to standing (or remain seated if not able to safely stand) - Mountain pose (standing) - Receptive gesture, cactus arms (shoulder flexion, shoulder extension, shoulder rolls, and arm movements) - Half moon (lateral flexion) - Seated savasana (body scan with progressive relaxation) |
|  |  |
| 3 | Last session plus:   - Intro to diaphragmatic breathing and extended exhalation - Move to standing (or remain seated if not able to safely stand) - Crescent moon (standing supported lunges) - Locust pose in standing (hip extension while standing) - Seated savasana (body scan with progressive relaxation) |
|  |  |
| 4 | Last session plus:   - Grounding (Centering) – connect to mantra and check in with body and emotions - Alternate nostril breathing - Intro to partner poses while seated– shoulder abduction and touching partner’s hand - Standing postures - Move to floor or chair - Intro to coming down to floor - Savasana on back or in chair |
|  |  |
| 5 | Last session plus:   - Partner pose – shoulder abduction, touching partner’s hand, adding silently saying the mantra (“I am strong. Together we are stronger”) - Standing postures - Move to floor or chair - Seated or supine pigeon (figure four) - Cactus arms - Savasana on back or in chair |
|  |  |
| 6 | Last session plus:   - Grounding (Centering) connect to mantra and check in with body and energy levels - Partner pose – (silently repeat mantra and visualize giving and receiving support with hand touching) - Standing postures - Move to floor - Bridge pose (supine hip extension) - Wind removing pose (hip and knee flexion to one leg at a time)   Savasana on back on in the chair |
|  |  |
| 7 | Last session plus:   - Partner pose – (mirroring technique seated across from each other and gazing into each other’s eyes while touching hands and silently repeating mantra) - Standing postures - Crescent moon (supported lunge) to Warrior I - Savasana on back or in chair |
|  |  |
| 8 | Last session plus Q/A and participant feedback |
|  |  |
